# Progress in Catalytic Pyrolysis of Oil Shale

**DOI:** 10.1155/2021/6759176

**Published:** 2021-10-07

**Authors:** Donghao Li, Haodan Pan, Xiaojing Di, Xiaoyang Liu, Hongxiang Hu

**Affiliations:** ^1^College of Petroleum Engineering, Liaoning Petrochemical University, Fushun 113301, China; ^2^Thermal Power Plant of PetroChina Fushun Petrochemical Company, Funshun 113301, China; ^3^CAS Key Laboratory of Nuclear Materials and Safety Assessment, Institute of Metal Research, Chinese Academy of Sciences, Shenyang 110016, China

## Abstract

This paper briefly describes the research status of oil shale pyrolysis technology and the main factors affecting oil shale pyrolysis, with emphasis on four kinds of commonly used catalysts: The effects of natural minerals, metal compounds, molecular sixes, and supported catalysts on the pyrolysis of oil shale were discussed. The changes of the pyrolysis mechanism and product composition of oil shale with the addition of different catalysts were discussed. Finally, the development direction of preparation of new catalysts was discussed, in order to provide a prospect for the development and utilization of unconventional and strategic alternative energy resources around the world.

## 1. Introduction

With the continuous decline of petroleum supply and the increase of petroleum product cost, how to solve the energy problem has become a big problem of urgency. Shale oil, the pyrolysis product of oil shale, is considered as a substitute for crude oil, and the utilization of this resource can alleviate the shortage of crude oil supply. Oil shale is an unconventional oil and gas resource, which is a nonrenewable energy source like oil, natural gas, and coal. It is rich in reserves and has great potential for industrial application [1]. The world is rich in oil shale reserves, up to 689 billion tons of shale oil. The United States has the largest oil shale reserves at 530.5 billion tons. China's reserves are 47.6 billion tons [2].

Because kerogen in oil shale is completely solid in its natural state, it cannot be directly mined. Kerogen in oil shale can be converted into liquid shale oil only by pyrolysis [2]. However, kerogen in oil shale conversion efficiency is low and affects the quality of shale oil, resulting in oil instability and high viscosity. Therefore, in order to promote the conversion of kerogen macromolecules, researchers used different types of catalysts to carry out catalytic pyrolysis, so as to improve the conversion efficiency of kerogen.

## 2. Research Status of Oil Shale Pyrolysis Technology

### 2.1. Application of Oil Shale Pyrolysis Technology

Oil shale pyrolysis technology mainly includes ectopic and in situ methods. Ectopic methods include oil shale extraction, grinding, screening, and pyrolysis. The in situ method involves heating and then pyrolyzing oil shale in geological formations [1, 2]. Therefore, many types of distiller have been derived in industry. Fushun distiller in China, Kiviter distiller in Estonia, and Petrosix distiller in Brazil have been successfully implemented and widely used [3].

Taking Fushun-type retorting technology as an example, the oil shale retorting process (OSR-GHC) with gas heat carrier has some disadvantages, such as low oil yield and low energy efficiency, which lead to low economic benefit [4]. Therefore, more and more attentions were paid to this technology of solid heat carrier retorting.

Different from the oil shale retorting process, the oil shale retorting process using solid heat carrier technology (OSR-SHC) uses a thermal cycle to provide heat for the oil shale retorting reaction. Compared with gas heat carrier retorting technology, the OSR-SHC process has many advantages: it can make the retorting air distribution uniform and reduce the flow resistance. Increase shale oil yield to 90%. Increase resource utilization rate, good economy, and reduce environmental pollution [5].

### 2.2. Factors Affecting Oil Shale Pyrolysis

The pyrolysis of oil shale is affected by many factors, among which the technological conditions (including pyrolysis temperature, residence time, heating rate, and pressure) have the most significant influence on its pyrolysis [6].

#### 2.2.1. Influence of Pyrolysis Temperature

The final pyrolysis temperature of oil shale has a complex influence on the pyrolysis process. The yield of shale oil, the composition of shale oil, the composition of pyrolytic semicoke, and secondary cracking of pyrolysis products are all related to the final pyrolysis temperature.

**Figure 1:**
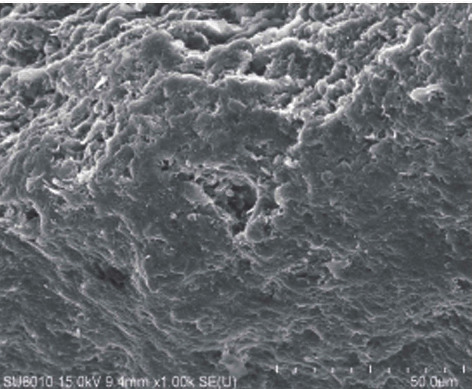
Pore size at 300°C.

**Figure 2:**
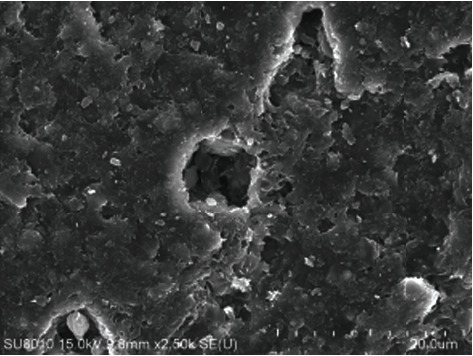
Pore size at 400°C.

**Figure 3:**
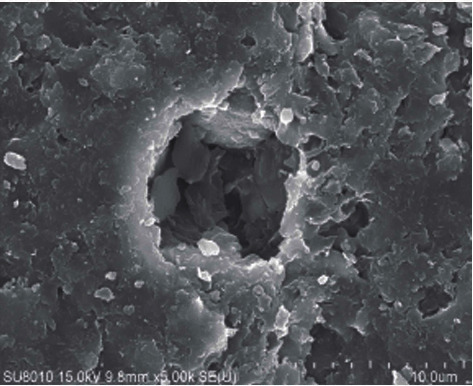
Pore size at 450°C.

**Figure 4:**
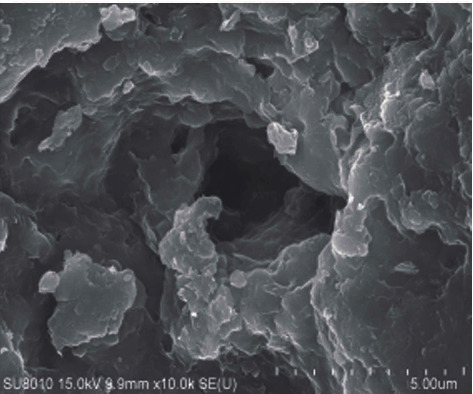
Pore size at 500°C.

Wang et al. [7] studied the effect of water vapor on the temperature in pyrolysis of oil shale as a heat-carrying fluid and found that between 382°C and 555°C, the permeability increased significantly with the increase of temperature, which was conducive to pyrolysis. Geng et al. [8] have studied the evolution of pore fractures using X-ray computed tomography (Figures 1–4). The results show that the porosity increases, and the degree of pyrolysis intensifies with the increase of temperature. From 300°C to 500°C, the most significant increases.

#### 2.2.2. Influence of Heating Time

Hydrothermal pretreatment affects the yield of oil shale pyrolysis products. The semicoke yield of oil shale increases, and the gas and water yield decreases. The shale oil yield can reach the maximum after 2 h hydrothermal pretreatment, and the oil produced from oil shale pyrolysis has higher energy [9]. Yang et al. [10] studied the influence of heat injection time on the quality of oil and gas products. When the injection temperature is controlled at 555°C, the quality of shale oil formed by oil shale cracking reaches the highest at 3 h of injection.

#### 2.2.3. Influence of Heating Rate

The effect of heating rate on shale oil production is small, corresponding to oil production in the range of 10.4-11%. With the acceleration of the heating rate, oil production increases while gas production decreases slightly [11]. Lu et al. [12] and Bai et al. [13] studied the pyrolysis process of oil shale. The influence of heating rate (5°C/min, 10°C/min, 20°C/min, and 50°C/min) was considered. The increase of heating rate can transfer the characteristic parameters of oil shale pyrolysis to the high temperature but has no effect on the total mass loss.

#### 2.2.4. Influence of Pressure

The influence of increasing pressure on the pyrolysis of organic matter depends on the amount of increasing pressure. Different increasing pressure values will have different effects and even block phenomena. The high-pressure thermogravimetric analysis of oil shale pyrolysis shows that the volatilization temperature of hydrocarbon increases with the increase of pyrolysis pressure. With the increase of pyrolysis pressure, oil production rate decreases, and gas production rate increases [14, 15].

## 3. Research Progress on Catalytic Pyrolysis of Oil Shale

Different parameters and conditions of catalytic pyrolysis of oil shale have been studied by using different catalysts. At present, the catalysts used in the catalytic pyrolysis of oil shale mainly include the following four types: natural minerals, metal compounds, molecular sieves, and supported catalysts.

### 3.1. Natural Minerals

As a kind of inorganic and organic sedimentary rock, the oil shale itself contains a variety of natural mineral components. Natural minerals such as quartz, clay minerals, feldspar, and pyrite are closely combined with organic matter in oil shale, which has a great influence on the pyrolysis process.

Oil shale is composed of organic and inorganic minerals. Inorganic minerals usually account for 50-85 wt% of oil shale, mainly including silicates, carbonates, quartz, and pyrite. Inorganic minerals have a certain influence on the pyrolysis of oil shale [16]. Zhao et al. [16] and Chang et al. [17] used acid to treat oil shale in batches in order to better understand the interaction between organic matter and inorganic minerals during oil shale pyrolysis. Hydrochloric acid effectively eliminates carbonate minerals, hydrofluoric acid effectively dissolves silicate minerals, and nitric acid removes pyrite. It was found that the shale oil production rates of original, carbonate-free, and carbonate-silicate oil shale samples were 50.4 wt.%, 44.3 wt.%, and 50.3 wt.%, respectively, indicating that carbonates promoted shale oil production and acted as catalysts in kerogen pyrolysis because their elimination from oil shale reduced hydrocarbon production [18]. The rate of shale oil production is reduced by silicates. This suggests that carbonates catalyze, sulfates catalyze, and the decomposition of kerogen also decreases with the increase of silicate. In addition, H_2_SO_4_ treatment can reduce the initial temperature and improve the pyrolysis efficiency of oil shale, which is economical [19].

Lu et al. [20] used HCL-HF to treat Huadian oil shale to better understand the interaction between organic matter and minerals in the pyrolysis process of oil shale. Mineral compounds have little effect on the decomposition of organic matter but have an effect on volatile matter reactions. It was found that CaCO_3_, kaolinite, and TiO_2_ had little effect on the volatiles reaction, while K_2_CO_3_, Na_2_CO_3_, and MnCO_3_ promoted the volatiles reaction. In the process of catalytic pyrolysis of oil shale, the alkyl side chains of carbonate are disconnected, more hydrocarbons and toluene are generated, and benzene and H_2_ are generated by dehydrogenation of long-chain aliphatic hydrocarbons. The action sequence of carbonate is K_2_CO_3_ > Na_2_CO_3_ > Mn_2_CO_3_.

The copyrolysis behavior of kerogen and montmorillonite showed that montmorillonite significantly improved the pyrolysis characteristics of kerogen. Therefore, montmorillonite can be considered as a potential natural catalyst [21]. Jiang et al. [22] studied the catalytic pyrolysis characteristics of oil shale mixed with montmorillonite and CoCl_2_·6H_2_O. It was found that montmorillonite and cobalt chloride promoted decarboxylation reaction, and free radical reaction decreased acid yield and increased aliphatic hydrocarbon yield. The relative content of aliphatic hydrocarbons increased from 41.55% to 51.27%. This indicates that the combined use of montmorillonite and cobalt chloride is beneficial to the improvement of pyrolysis characteristics of oil shale and the formation of low molecular weight hydrocarbon fuels. Moreover, with the increase of montmorillonite ratio, shale oil yield increases, and the presence of cobalt chloride further promotes the deep pyrolysis of oil shale. When the cobalt chloride/montmorillonite mass ratio was 1 ∶ 5, the maximum yield of liquid fuel increased by about 3.5 wt%.

Shi et al. [23] studied the influence of shale ash on the oil shale pyrolysis process. It is found that shale ash has little effect on product yield, but a great effect on gas and oil composition. Under the action of shale ash, the production of H_2_ and CH_4_ increased, but CO_2_ decreased significantly. It reduces the aliphatic content in shale oil, shortens the chain length of the aliphatic group, but promotes the formation of aromatic hydrocarbon. This effect increases with the increase of shale ash content. Shale oil production is rising.

### 3.2. Metal Compounds

Metal compounds including metal oxides, metal sulfides, and metal salts have the advantages of simple preparation and high activity. Therefore, the effect of metal compounds on the yield and quality of liquid products has been studied extensively. Studies have found that Fe, Ca, Zn, Ni, and other metal oxides and chloride can accelerate the pyrolysis of oil shale and promote the generation of hydrogen-free radicals and the lightening of shale oil [24].

After the Fe_2_O_3_ catalyst was added and the CaCO_3_ catalyst was added, the shale oil yield also increased in the pyrolysis process, but the shale oil yield was 1.02 times and 1.01 times, respectively. Therefore, Fe_2_O_3_ has a stronger catalytic effect on oil shale pyrolysis [24]. Lu et al. [12] selected CH_3_COONa, (CH_3_COO)_2_Ca, and MgO as oil shale catalysts to study their pyrolysis behavior and characteristics of pyrolysis products. The results show that the three catalysts can improve the shale oil and gas production rate. These catalysts redirect the pyrolysis process and improve the yield of shale oil and pyrolysis gas. CH_3_COONa and MgO make the precipitation temperature of oil shale lower than that of the original sample, thus, reducing the activation energy of oil shale pyrolysis. Kang et al. [25] and Kang et al. [26] extracted Huadian oil shale and added different Fe compounds. After adding FeCl_3_, the yield of shale oil from oil shale pyrolysis within 20 hours increased by 58.5%, and the time required for shale oil to produce the maximum oil decreased by 43%. This suggests that FeCl_3_ accelerates the decomposition of asphaltenes in residual bitumen by promoting the cleavage of heterotopic bonds and triggering the pyrolysis reaction of kerogen. The presence of FeCl_3_ inhibited the condensation polymerization of kerogen and promoted the ring-opening reaction of the aromatic structure. At 350°C, within 20 hours after FeCl_2_ was added, the concentration of asphalt obtained was 0.08 mol/L, 50.5% higher than that of pure water. The addition of FeCl_2_ reduced the time required to reach the maximum yield of bitumen by 43%. The results show that FeCl_2_ solution extraction is an efficient method for in situ extraction of oil shale.

Jiang et al. [27] and Chang et al. [28] studied the effects of several transition metal salts such as FeCl_2_·4H_2_O, CoCl_2_·6H_2_O, NiCl_2_·6H_2_O, MnSO_4_·H_2_O, and ZnCl_2_ on oil shale pyrolysis based on thermal decomposition characteristics, product yield, and composition of oil shale. It is concluded that these metal salts can promote the secondary cracking of shale oil, reduce oil production, and increase the production of pyrolysis gas. Metal salts can also catalyze the aromatization of aliphatic hydrocarbons to produce aromatic hydrocarbons. When MnSO_4_·H_2_O and CoCl_2_·6H_2_O are loaded with 0.1%MnSO_4_·H_2_O and CoCl_2_·6H_2_O, the initial precipitation temperature of oil shale is lower than that of the original sample. In the second stage of pyrolysis (430~520°C), the activation energy decreases by 3.621 kJ/mol and 5.964 kJ/mol, respectively, and the oil yield increases by 0.44% and 0.53%, respectively, at 520°C. Meanwhile, NiCl_2_·6H_2_O can also promote oil shale pyrolysis. FeCl_2_·4H_2_O and ZnCl_2_ have little effect on the decomposition behavior of oil shale. Metal salts can also catalyze the aromatization of aliphatic hydrocarbons to produce aromatic hydrocarbons.

### 3.3. Molecular Sieve

Molecular sieve has a special pore structure and is widely used in the chemical process industry as a new catalyst. The commonly used molecular sieves are mainly SAPO-11 molecular sieve, aluminum phosphate molecular sieve, ZSM-5 molecular sieve, TS molecular sieve, MCM molecular sieve, SBA molecular sieve, etc.

In addition, the molecular sieve can be entered according to the size of the order, but also has the advantages of uniform aperture structure, large specific surface area, high surface polarity, and stable structure. If the metal ions are doped in the molecular sieve, the acidity and REDOX characteristics will be affected. MCM-41 has a catalytic effect on the pyrolysis of oil shale and is widely used in hydrocarbon conversion processes, including aromatics dealkylation, cracking, and hydrocracking. MCM-41 has a unique cracking selectivity that can increase shale oil production [29].

ZSM-5 zeolite catalyst was added into the reactor to process the steam generated from the pyrolysis of oil shale. The effects of zeolite catalysts on the yield and composition of derived oil and gas were studied. The main gases produced by the pyrolysis of oil shale are carbon dioxide, carbon monoxide, hydrogen, methane, C_2_H_4_, C_2_H_6_, C_3_H_6_, and a small number of other hydrocarbon gases. After catalysis, the concentration of all hydrocarbon gases increased. But the oil yield is reduced, which leads to higher gas yield and coke formation on the catalyst. The total nitrogen and sulfur contents in oil were significantly reduced by 67% for nitrogen and 56% for sulfur [30]. Chang et al. [31] conducted catalytic pyrolysis of Huadian oil shale using ZSM-5 (SAR = 25, 38, 50) as a catalyst. Pyrolysis experiments were carried out in ZSM-5 (10 wt%) oil shale. The results showed that the ZSM-5 catalyst reduced the shale oil yield from 9.33 wt% to 6 wt%. The content of aromatic hydrocarbons in shale oil increased from 2.88% to more than 20%. The contents of aromatic hydrocarbons of ZSM-5-25 and ZSM-5-38 are 59.39% and 56.46%, respectively, which are suitable catalysts for catalytic pyrolysis. Compared with ZSM-5-38, ZSM-5-25 catalyzed less monocyclic aromatic hydrocarbons and more polycyclic aromatic hydrocarbons.

Park [32] studied the pyrolysis and catalytic pyrolysis of black pine (BPW) and cook oil shale (KOS) on acidic zeolite by gas chromatography-mass spectrometry. The results of gas chromatography-mass spectrometry (GC-MS) showed that oxygen-containing compounds and light hydrocarbons were generated from black pine and cook oil shale during noncatalytic pyrolysis. These oxygen-containing compounds and light hydrocarbons are converted to aromatic hydrocarbons over acidic zeolite by catalytic pyrolysis. The catalytic pyrolysis efficiency of BPW and KOS on HZSM-5 is up to 56%.

### 3.4. Supported Catalysts

Load type catalyst consists of active component and carrier of two parts; the load on the carrier was promoted after the dispersion of active components and can reduce the dosage; the carrier who is usually a big specific surface area and thermal stability of good material, such as natural ores and molecular sieve; and they can not only separate as a catalyst but also can be the carrier of catalyst. The active ingredients are mainly composed of one or several kinds of Fe, Co, Mo, Ni, etc. The supported catalyst support and active component also show a synergistic effect in some specific reactions.

Shale ash has a catalytic effect on oil shale pyrolysis and has a good adsorption capacity. As the grain size of shale ash decreased from 1.25 mm to 0.20 mm, the shale coke yield decreased slightly and the total volatile product yield increased. The addition of 0.20 mm shale ash is the best for improving shale oil production rate and kerogen conversion to volatile products [33]. Lu et al. [34] studied the effect of Fushun oil shale and shale ash loaded with different transition metal salts (ZnCl_2_, NiCl_2_·6H_2_O, and CuCl_2_·2H_2_O) on pyrolysis. The transition metal salt catalyst supported by shale ash can reduce the initial pyrolysis temperature of oil shale, and the catalytic effect is enhanced with the increase of transition metal salt loading in the range of 0.1-3.0 wt%. Different transition metal salts have different catalytic effects. CuCl_2_·2H_2_O has the most obvious catalytic effect among the three excessive metal salts. The loading of transition metal salts on shale ash not only increases the content of oxygen-containing compounds but also promotes the cracking and aromatization of aliphatic hydrocarbons to form short-chain aliphatic hydrocarbons and aromatic hydrocarbons. The catalytic sequence of transition metal salts is CuCl_2_ · 2H_2_O > NiCl_2_ · 6H_2_O > ZnCl_2_. Liu et al. [35] studied using shale ash (SA) as support, Cu-Ni transition metal salts with different ratios of Cu/Ni are 1 : 0, 2 : 1, 1 : 1, and 1 : 2, 0 : 1 to investigate the effects of different ratios of transition metal salts on the pyrolysis behavior and characteristics of Fushun oil shale. The results show that the temperature corresponding to the maximum weight loss rate decreases by 12.9°C, 4.0°C, and 3.6°C, respectively, and the apparent activation energy of pyrolysis decreases by 35.2%, 33.9%, and 29.6% with the addition of Cu_0_Ni_1_/SA catalyst, respectively. The addition of Cu_0_Ni_1_/SA and Cu_2_Ni_1_/SA further improved the shale oil yield by 3.5% and 3.1%, respectively.

After hydrodesulfurization, the calorific value and viscosity of shale oil are improved obviously. Catalysts used for modification are always inactivated by coke deposition, but in situ high-temperature treatment in the air effectively removes the coke from the catalyst surface to make it highly active after the devulcanization operation. Zhang et al. [36] and Qiao et al. [37] used Ni-Mo/Al_2_O_3_ catalyst to conduct fixed-bed mild hydrotreating for shale oil. The results show that under the condition of catalytic hydrodesulfurization, the sulfur removal rate of shale oil is 84.6%, and the yield of upgraded high-quality oil is up to 96.2%. After hydrodesulfurization, the calorific value and viscosity of shale oil are improved obviously.

## 4. Prospect of Catalytic Pyrolysis Technology of Oil Shale

Researchers have carried out a large number of studies on various factors affecting oil shale pyrolysis and achieved fruitful results. The optimum conditions involving pyrolysis temperature, heating rate, residence time, and pressure have been determined.

The future research direction of oil shale pyrolysis will focus on catalysts: (1) further research on alkali metal, alkaline earth metal, and molecular sieve catalysts; (2) the supported catalyst was designed with molecular sieve, montmorillonite, and Al2O3 as the carrier; (3) the corresponding catalysts were designed by deeply understanding the macroscopic and microscopic structural characteristics of oil shale in different areas, and the catalytic cracking mechanism was studied. At present, there have been researches on binary supported catalysts based on single active component supported catalysts, but there are still few types of research in this aspect. In the future, with further research, there will be more binary or even multiple supported catalysts or more complex catalysts to promote the development and progress of the oil shale industry.

## Data Availability

The review article's data used to support the findings of this study are included within the article.
